# The multiple functional roles of mesenchymal stem cells in participating in treating liver diseases

**DOI:** 10.1111/jcmm.12482

**Published:** 2014-12-23

**Authors:** Wei-hui Liu, Fu-qiang Song, Li-na Ren, Wen-qiong Guo, Tao Wang, Ya-xing Feng, Li-jun Tang, Kun Li

**Affiliations:** a General Surgery Center, Chengdu Military General HospitalChengdu, Sichuan Province, China; b Experimental Medical Center, Chengdu Military General HospitalChengdu, Sichuan Province, China; c Nursing College, Chengdu Medical SchoolChengdu, Sichuan Province, China

**Keywords:** mesenchymal stem cells, transdifferention, cell fusion, hepatocytes, paracrine effect

## Abstract

Mesenchymal stem cells (MSCs) are a group of stem cells derived from the mesodermal mesenchyme. MSCs can be obtained from a variety of tissues, including bone marrow, umbilical cord tissue, umbilical cord blood, peripheral blood and adipose tissue. Under certain conditions, MSCs can differentiate into many cell types both *in vitro* and *in vivo*, including hepatocytes. To date, four main strategies have been developed to induce the transdifferentiation of MSCs into hepatocytes: addition of chemical compounds and cytokines, genetic modification, adjustment of the micro-environment and alteration of the physical parameters used for culturing MSCs. Although the phenomenon of transdifferentiation of MSCs into hepatocytes has been described, the detailed mechanism is far from clear. Generally, the mechanism is a cascade reaction whereby stimulating factors activate cellular signalling pathways, which in turn promote the production of transcription factors, leading to hepatic gene expression. Because MSCs can give rise to hepatocytes, they are promising to be used as a new treatment for liver dysfunction or as a bridge to liver transplantation. Numerous studies have confirmed the therapeutic effects of MSCs on hepatic fibrosis, cirrhosis and other liver diseases, which may be related to the differentiation of MSCs into functional hepatocytes. In addition to transdifferentiation into hepatocytes, when MSCs are used to treat liver disease, they may also inhibit hepatocellular apoptosis and secrete various bioactive molecules to promote liver regeneration. In this review, the capacity and molecular mechanism of MSC transdifferentiation, and the therapeutic effects of MSCs on liver diseases are thoroughly discussed.

- Introduction
- Sources of MSCs for treating liver disease
- Characterization of HLCs differentiated from MSCs
- Modulation of MSC transdifferentiation into hepatocytes
  – Cytokines and growth factors
  – ECM cues
  – Modification of the physical parameters in MSCs cultures
- Mechanisms of MSCs transdifferentiation into hepatocytes
  – Transcription factors determine the transdifferentiation of MSCs
  – Cellular signalling pathways control the transdifferentiation of MSCs
  – Epigenetic modification affects the transdifferentiation of MSCs
  – Mesenchymal-epithelial transition accompanies the transdifferentiation of MSCs
- The potential strategies of MSC-mediated therapy on liver disease
  – Transdifferentiation and cell fusion to restore damaged liver
  – Immunomodulatory effects of MSCs to repress immune destruction
  – Paracrine effect of MSCs
  – MSC inhibits hepatocellular apoptosis and stimulates liver regeneration
- Problems and perspectives

## Introduction

There are many causative factors (toxic injury, viral infections, autoimmune defects, genetic disorders) that can cause liver dysfunction, such as chronic liver disease or acute liver failure. Liver transplantation remains the primary treatment for end-stage liver diseases. However, the main limitation of this treatment is the shortage of donor organs. Moreover, adverse factors, such as rejection, inevitable side-effects associated with the long-term use of immunosuppressants and high cost, make liver transplantation unfavourable for many patients 1,2.

In view of the above shortcomings, cell-based hepatocyte and bioartificial liver transplantation have developed into alternative approaches for the treatment of liver failure because of the use of simpler and less-invasive procedures 3,4. By doing so, a single donor could serve several patients, and excess cells could be cryopreserved for future use. Unfortunately, the low cell viability and instability of transplantable hepatocytes has hampered their clinical application. Studies have shown that less than 30% of transplanted hepatocytes survive *in vivo*. Meanwhile, the surviving cells have limited replicative potential and loss of basic hepatic function as a result of culture *in vitro* 4,5.

In the search for an ideal cell resource for the treatment of liver diseases, mesenchymal stem cells (MSCs) have attracted considerable attention. Compared to organ and hepatocyte transplantation, MSC transplantation mainly has the following advantages. First, MSCs are not derived from somatic cells and are superior in terms of ethical concerns related to the treatment of liver diseases. Second, MSCs can be obtained with relative ease and expanded in culture. These cells are readily available from a variety of tissues, such as bone marrow (BM), umbilical cord blood (UCB), peripheral blood, the synovial membrane and adipose tissue (AT). Third, MSCs can differentiate into a wide variety of cell types. In the past decade, large studies have shown that under certain defined conditions, both rodents and human MSCs can differentiate into hepatocyte-like cells (HLCs) possessing the functions of adult hepatocytes 6,7. In addition, studies suggest that MSCs have low inherent immunogenicity and are capable of modulating immunological responses through interaction with various immune cells, improving the safety of using MSCs. MSCs have been shown to be a promising candidate for liver regeneration, and numerous applications have been developed. In this survey, we review landmark studies on this topic, analyse the key factors that influence the differentiation of hepatocytes from MSCs, discuss the mechanisms of the differentiation of MSCs into hepatocytes and summarize the therapeutic effects of MSCs on liver diseases.

## Sources of MSCs for treating liver disease

**Fig 1:**
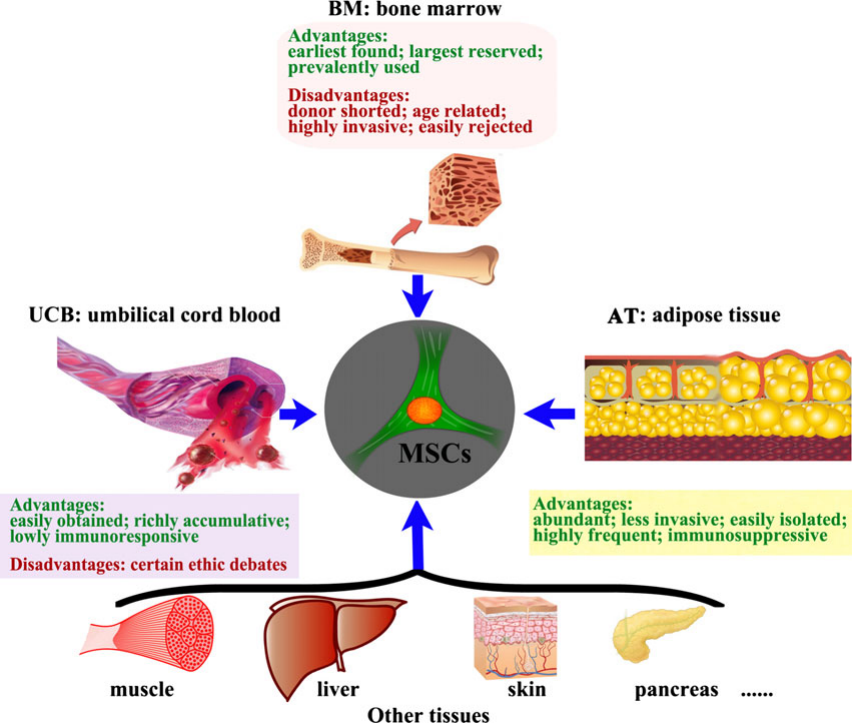
Sources of MSCs and the characteristics of MSCs derived from the three main sources for treating liver disease. Abbreviation: MSCs, mesenchymal stem cells.

Mesenchymal stem cellhave been described as non-hematopoietic, undifferentiated, fibroblast-like, multipotent progenitor cells with the capacity to differentiate into multiple mesenchymal cell lineages, such as osteoblasts, chondrocytes and adipocytes 8. MSCs can be isolated from most organs or tissues, including the BM, AT, UCB, peripheral blood, trabecular bone, synovial membrane, cartilage and muscle 9–11. In this section, we discuss the three main sources of MSCs that have been demonstrated to possess the ability to treat liver disease, including BM-MSCs, AT-MSCs and UCB-MSCs (Fig.1).

Recent studies in rats 12, mice 13 and humans 14 confirmed that BM-MSCs, UCB-MSCs or AT-MSCs can differentiate into HLCs under selective growth conditions *in vitro*. In addition to their hepatic differentiation potency, MSCs can produce a series of cytokines and signalling molecules that can have pleiotropic effects at the site of liver lesions, including immune-modulatory, anti-inflammatory, anti-apoptotic, and pro-proliferative effects 15. More importantly, MSCs are not derived from somatic cells and are superior in terms of ethics and safety in the treatment of liver diseases. All of these features make MSCs an ideal cell resource for the treatment of a variety of liver entities.

Interestingly, three kinds of above mentioned MSCs have different benefits and drawbacks in treating liver disease. Kern *et al*. compared MSCs derived from different sources with regard to morphology, isolation success rate, frequency of colony formation, expansion potential, multipotency and immune phenotype 16. Generally speaking, there are no significant differences concerning the morphology and immune phenotype of the MSCs derived from these three sources 16. In detail, BM is the largest reservoir of MSCs among various tissues and BM-MSCs are the most prevalently used cell type. However, BM may be detrimental for clinical use because of the highly invasive donation procedure and the decline in MSC number and differentiation potential with increasing age. More recently, UCB-MSCs, which can be obtained using less-invasive methods, were introduced as an alternative source of MSCs 11. UCB-MSCs are not being harvested from the newborns, easy to obtain, available for collection after delivery and remain viable after long-term cryopreservation. Another promising source is AT. AT-MSCs have the relative advantages of accessibility, abundance and immunosuppressive properties 17. In fact, compared with MSCs from other sources, AT-MSCs possessed the highest proliferation capacity, they were the most abundant, they could be isolated using a less-invasive procedure, and they were easy to harvest by simple lipoaspiration 18. Nevertheless, the choice of MSCs should be more related to the function and repair potentiality for the liver. That means the chosen source of MSC should have the best differentiation potential into hepatocytes and/or highest paracrine effect for the injured liver repair, than other sources of MSCs. According to the above criteria, BM-MSCs still seem to be the best choice so far.

## Characterization of HLCs differentiated from MSCs

**Fig 2:**
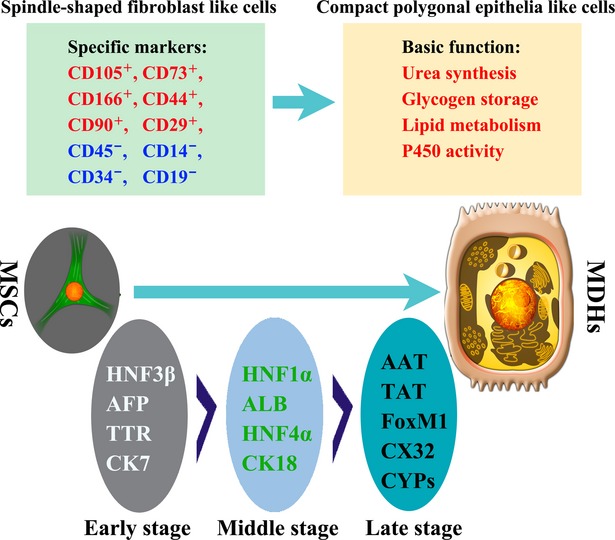
Characterization of MSCs during differentiation into MDHs. Changes occur when MSCs differentiate into MDHs, including alteration of the morphological, phenotypic and functional characteristics. Abbreviations: MSCs, mesenchymal stem cells; MDHs, MSC-derived hepatocytes.

MSCs are defined as plate-adhering, fibroblast-like cells possessing the ability for self-renewal and the capacity to differentiate into multiple mesenchymal cell lineages such as osteoblasts, chondrocytes and adipocytes 8. Meanwhile, MSCs express surface markers, such as CD105, CD73 and CD90, and lack of expression of CD45, CD14 and CD34 or CD11b and CD79a or CD19 and HLA-DR surface molecules 19. The characterization of MSCs during differentiation into MSC-derived hepatocytes (MDHs) is of great importance and generally includes morphological, phenotypic and functional characterization (Fig.2).

Commonly, with regard to the morphology of MSCs, under conditions favoring hepatic differentiation, a change from a fibroblast-like morphology to the polygonal shape typical of epithelial cells can be observed. For example, Pournasr *et al*. 20 demonstrated during the initiation step of hepatic differentiation from human MSCs, the cells showed a remarkable transition from a bipolar fibroblast-like morphology to a round epithelial cell-like shape from days 6–7. At this time, the cells were still surrounded by spindle-shaped cells. The contraction of the cytoplasm progressed further as maturation continued. Meanwhile, most cells became quite dense and round with clearly double nuclei in the late differentiation stage. Small round or oval-shaped cells with a polyhedral structure were visualized from days 14–40, which was similar to primary hepatocytes that underwent morphological changes during hepatic induction.

Phenotypically, several liver transcription factors and cytoplasmic proteins were selectively expressed during the differentiation of MSCs. For human, during the early stage of MSC-to-hepatic differentiation, these cells express early markers, such as hepatocyte nuclear factor (HNF)3β, alpha-fetoprotein (AFP) and transthyretin (TTR), followed by the expression of mid/late markers [HNF1α, HNF4α, albumin (ALB) and cytokeratin (CK) 18] 21,22. Then, during the late stage of MSC differentiation, they express cell markers and proteins similar to mature hepatocytes, such as tryptophan 2,3-dioxygenase (TO), anti-trypsin (AAT), tyrosine amino transferase (TAT), CCAAT-enhancer-binding protein (C/EBP) α, forkhead transcription factor (FoxM1), hepatocyte-specific gap junction protein (CX32) and cytochrome P450 (CYPs) 21,22. At the same time, differentiated hepatocytes gradually lose the expression of mesenchymal cell markers such as α-Actin(α-SMA)^21^,^22^. In general, the most well-studied markers include the transcription factors (HNF1α/β, HNF3β, HNF4α and C/EBPα/β), plasma proteins (AFP, ALB, TTR) and cytoskeletal proteins (CK18, CK8) 21,22.

The major functions of the liver include glycogen storage, detoxification and lipid metabolism. Common tests of liver function, such as glycogen deposition, detoxification of ammonia through the synthesis of urea, the uptake of low-density lipoprotein and phenobarbital-inducible cytochrome P450 activity, have been widely used to test the function of MDHs 22. Other tests, such as assays for cellular glutathione (GSH) and glutathione S-transferase (GST) activity, are also used to discriminate MDHs from undifferentiated MSCs 23.

## Modulation of MSC transdifferentiation into hepatocytes

**Fig 3:**
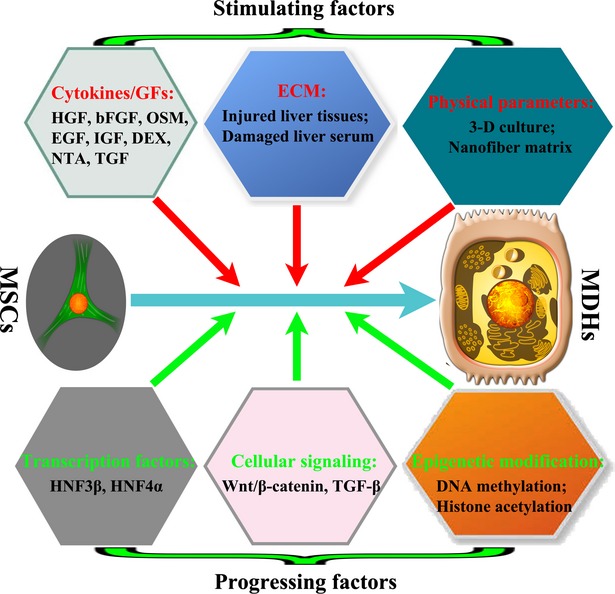
The modulation of MSC differentiation into hepatocytes. The modulation of MSC differentiation into MDHs can be induced by various factors. Extracellular (stimulating) factors: here we mainly discuss the roles of cytokines, growth factors, ECM cues and the physical parameters of culture. Intracellular (progressing) factors: here we focus on three key players involved in the differentiation of MSCs: transcription factors, cellular signalling and epigenetic modification. Abbreviations: MSCs, mesenchymal stem cells; MDHs, MSC-derived hepatocytes; ECM, extracellular matrix; GFs, growth factors.

The high degree of plasticity of MSCs has been widely demonstrated during the last decade. In this section, we discuss the following key points that influence the hepatic differentiation of MSCs: (*i*) cytokines and growth factors; (*ii*) cues from the extracellular matrix (ECM) and (*iii*) physical parameters for MSC culture. Understanding the influence of these factors will aid in the development of improved strategies for inducing the hepatic differentiation of MSCs (Fig.3).

### Cytokines and growth factors

Previous reports support the idea that liver injury or culture with foetal liver-conditioned medium can induce the differentiation of MSCs into functional hepatocytes 24,25. Differentiation is thought to be induced by cytokines secreted from the injured liver cells because no cellular interactions occur in cell-free cultural medium. However, which cytokines direct the specification of a hepatic fate remains unclear.

As we know, many cytokines and growth factors have certain effects on liver cell growth and differentiation *in vitro* 26, including hepatocyte growth factor (HGF), oncostatin M (OSM), epidermal growth factor (EGF), transforming growth factor beta (TGF-β), basic fibroblast growth factor (bFGF), insulin-like growth factor (IGF), dexamethasone (DEX) and nicotinamide (NTA). Among these factors, HGF, bFGF and OSM are particularly important for hepatic differentiation of MSCs. HGF and bFGF play essential roles in the development and regeneration of the liver, especially in the early stages of hepatogenesis 27. The results demonstrated that FGF-4, HGF and OSM were key cytokines involved in hepatic differentiation of MSCs 25. In addition to these three key cytokines, other factors are also important for the differentiation of MSCs into hepatocytes, such as IGF-1, NTA and DEX. IGF-1 can promote the proliferation of primary hepatocytes 28, NTA and DEX can introduce the proliferation of primary hepatocytes 29.

It should be mentioned that using a two-step procedure to induce hepatic differentiation from human BM-MSCs and UCB-MSCs, Lee 14 obtained functional HLCs *in vitro*. Prior to induction with this two-step procedure, cells from the 5th to 13th passage were deprived of serum for 2 days in Iscove's modified Dulbecco's medium (IMDM) supplemented with 20 ng/ml EGF and 10 ng/ml bFGF. Then, differentiation was induced by treating the MSCs with step-one differentiation medium, consisting of IMDM supplemented with 20 ng/ml EGF and 10 ng/ml bFGF, 0.61 g/l NTA, for 7 days, followed by treatment with step-two maturation medium, consisting of IMDM supplemented with 20 ng/ml OSM, 1 μmol/l DEX and 50 mg/ml premixed insulin-transferrin-selenium (ITS). Through this two-step procedure, their group obtained differentiated cells with characteristics of hepatocytes, including ALB production, glycogen storage, urea secretion, uptake of low-density lipoprotein and phenobarbital-inducible CYP450 activity.

### ECM cues

Treatment of the liver with CCl_4_ leads to the loss of a considerable amount of parenchyma tissue near the centrilobular regions as a result of necrosis. This necrotic tissue is cleared by immune cells, leaving the ECM components. The liver parenchyma then regenerates on this remnant matrix, over the damaged area. Interestingly, the damaged area, which was surrounded by ECM, was also the region that allowed engraftment and differentiation of MSCs 30.

The importance of the liver ECM for differentiation of MSCs has been recognized recently. The lack of ECM cues in a healthy liver resulted in a failure to trigger the differentiation of MSCs. Consistent with this, it was shown that BM-MSCs can differentiate into HLCs either in the presence of damaged liver-conditioned medium or medium supplemented with serum from individuals with liver damage 31. The pretreatment of MSCs with injured liver tissue represents a novel strategy to augment the differentiation ability of cells towards hepatic lineages. Furthermore, the pretreated MSCs demonstrated better survival, proliferation, differentiation and functional abilities 25. These findings provide useful information for the differentiation of MSCs towards a hepatic lineage. However, the mechanism has not been fully elucidated, and it could be related to the signalling molecules released into the ECM or the bloodstream in response to liver injury. Studies have shown that there are many cytokines and growth factors present in liver tissue following CCl_4_-induced damage, such as HGF, OSM, FGF4 and VEGF 32.

### Modification of the physical parameters in MSCs cultures

The use of a three-dimensional (3D) culture system can improve the preservation of membrane polarity and cell structure, allowing the tissue to maintain its functional properties 33,34. Research related to the use of 3D culture for hepatic differentiation of MSCs suggested that the physical characteristics of the culture can influence cellular phenotype and function. Similar to the study of Lin *et al*. 35, our group used a 3D alginate scaffold to culture MSCs and found that the 3D scaffolds were highly biocompatible with BM-MSCs and induced their differentiation into HLCs 36. Hashemi *et al*. 37 showed that UCB-derived MSCs differentiated on poly (ε-caprolactone) nanofibers and expressed several HLC markers. Piryaei *et al*. 38 demonstrated that the topographical properties of nanofibers enhance the differentiation of HLCs from MSCs and helps them to maintain their function in long-term culture. These observations revealed that not only are the dimensions of the topographical features important but their conformation, such as the presence of ridges, whorls, pores and grooves, even their symmetry can also influence the features of MSCs.

## Mechanisms of MSCs transdifferentiation into hepatocytes

The efficiency of hepatic differentiation of MSCs has been improved by modifying culture conditions or by adding various growth factors and cytokines. However, the efficiency of hepatic differentiation from MSCs is still insufficient for clinical application. Further investigation of the regulatory factors and the mechanisms of differentiation will be necessary to improve the efficiency of transdifferentiation of MSCs into hepatocytes (Fig.3).

### Transcription factors determine the transdifferentiation of MSCs

To understand the mechanisms underlying the hepatic differentiation of MSCs, several nuclear factors were investigated. For example, HNF3β and HNF4α were shown to play an important role. HNF3β is a member of the forkhead box transcription factor family and is thought to be a key player in hepatogenesis because it directly regulates expression of a number of hepatocyte-specific genes, such as AFP, ALB and TAT 39. Ishii *et al*. 40 established a tetracycline (Tet)-regulated expression system for HNF3β and demonstrated that HNF3β activation significantly enhanced the expression of ALB, AFP, TAT and epithelial cellular adhesion molecule. During treatment with the Tet-on system for 8 days, over 80% of the cells expressed ALB, indicating that HNF3β induces efficient differentiation of MSCs.

Furthermore, HNF4α plays a crucial role in the development of the liver-specific phenotype through the induction of various liver specific functions 41. Several studies have demonstrated that HNF4α may act as a master gene in a transcription factor cascade that could drive hepatic differentiation 42. These studies suggest that high expression of HNF4α may be a simple mechanism for the induction of hepatic differentiation and the function of HLCs derived from stem cells 43. Their findings indicate that HNF4α also plays a key role in facilitating the hepatic differentiation of MSCs.

### Cellular signalling pathways control the transdifferentiation of MSCs

Until now, the molecular signals regulating the transdifferentiation of MSCs have not been fully characterized. The Wnt family is essential for hepatic embryogenesis and is implicated in hepatic carcinogenesis. In recent years, Wnt signalling has also been shown to play major roles in self-renewal as well as the differentiation of MSCs 44. Ishii *et al*. 40 demonstrated that the down-regulation of Wnt/β-catenin signalling caused by the translocation of β-catenin to the cytoplasmic surface of the plasma membrane is associated with hepatic differentiation of human BM-MSCs. Meanwhile, these investigators also found that in human UCB-MSCs 44, the down-regulation of Wnt/β-catenin signalling with small interfering RNA also enhanced the hepatic differentiation of these cells. These studies suggest that Wnt/β-catenin signalling plays an important role in the hepatic differentiation of human MSCs and that the inhibition of Wnt signalling can promote the differentiation of MSCs into hepatocytes.

### Epigenetic modification affects the transdifferentiation of MSCs

Epigenetic modifications, such as DNA methylation and histone acetylation, may also contribute to the differentiation of MSCs. Snykers *et al*. 45 found that the addition of trichostatin A (a chromatin remodelling agent) to cultures of human MSCs that had been pretreated with hepatogenic agents for 6 days stimulated their transdifferentiation into cells with phenotypic and functional characteristics similar to those of primary hepatocytes. Furthermore, a microarray-based integrated analysis of methylation using isoschizomers was performed for genome-wide profiling of the DNA methylation status of AT-MSCs and AT-MDHs 46. Although the relationship between the altered DNA methylation status and hepatic differentiation is not completely understood, these genome-wide methylation patterns will also help to clarify the mechanism of hepatic differentiation from MSCs.

### Mesenchymal-epithelial transition accompanies the transdifferentiation of MSCs

Mesenchymal-epithelial transition (MET) and the reverse, epithelial-mesenchymal transition (EMT) are key developmental programs that play fundamental roles in the differentiation of multiple tissues and organs during embryogenesis 47 and carcinoma progression 48. Using microarray, Yamamoto *et al*. 49 compared the gene expression profiles of three populations: undifferentiated AT-MSCs, AT-MDHs and human primary hepatocytes. The results indicated that the expression levels of Twist and Snail, which are regulators of EMT, were down-regulated during the differentiation process. Furthermore, epithelial markers, such as E-cadherin and α-catenin, were up-regulated in AT-MDHs. Meanwhile, the expression of mesenchymal markers, such as N-cadherin and vimentin, was down-regulated. These findings suggest that MET occurs in the process of hepatic differentiation from AT-MSCs. In subsequent studies, in addition to Twist and Snail, several members of the TGF-β (SMAD7, LTBP2) and Wnt (FZD4, FZD6, DKK3) signalling pathways, along with the transcription factors that regulate these cellular cascades TCF7L1 and ID1 50, were found to be important in the process of hepatic differentiation from MSCs. Altogether, these data imply that cellular plasticity observed in MSCs is dependent on the MET 51.

## The potential strategies of MSC-mediated therapy on liver disease

**Fig 4:**
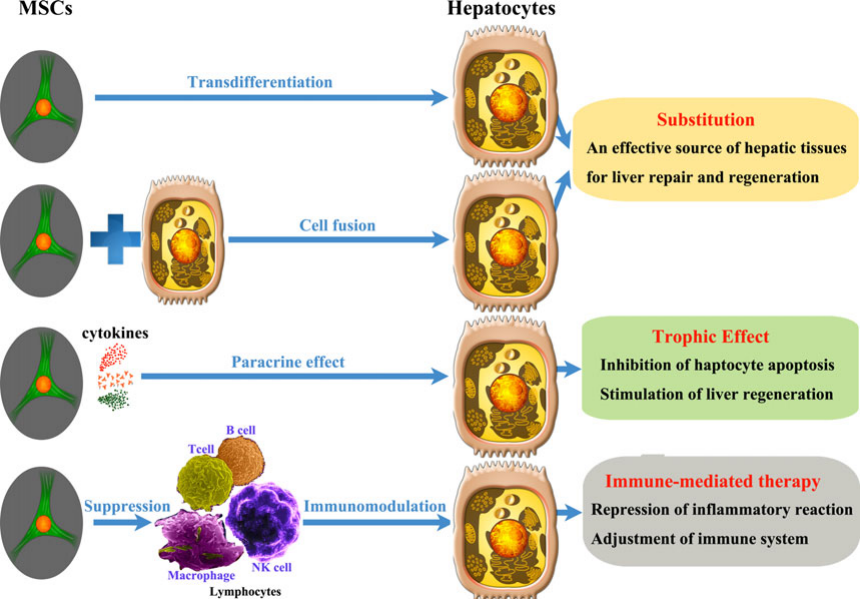
The therapeutic effects of MSCs in liver disease. The therapeutic effects of MSCs in liver disease can be attributed to three features. First, MSCs can serve as a substitute for functional hepatocytes through transdifferentiation or by cell fusion to repair liver tissue. Second, MSCs synthesize a wide variety of growth factors and cytokines and may exert a paracrine effect to enhance the repopulation of endogenous cells in necrotized tissue. Third, MSCs exert a generally suppressive effect on a wide variety of cells, including T and B lymphocytes and natural killer cells (NKs), to provide an immunomodulatory effect in immune-mediated liver diseases.

Considerable research has been performed on the role of MSCs in the treatment of liver disease. We attribute the therapeutic effects of MSCs on liver disease to the following aspects. First, MSCs have the ability to differentiate into or fuse with hepatocytes when injected into injured liver tissues, serving as an effective resource for hepatic tissue repair and regeneration. Second, MSCs can exert a generally suppressive effect on a wide variety of cells, including T and B lymphocytes and natural killer cells (NKs). This immunomodulatory effect provides a rational basis for the application of MSCs in the treatment of immune-mediated diseases. Third, MSCs synthesize a wide variety of growth factors and cytokines, and they may exert a paracrine effect to promote the repopulation of endogenous cells in necrotized tissue. Meanwhile, MSC-conditioned medium (MSC-CM) can also inhibit hepatocellular apoptosis and stimulate liver regeneration (Fig.4).

### Transdifferentiation and cell fusion to restore damaged liver

Cell fusion and direct differentiation (transdifferentiation) are the two possible pathways by which MSCs can achieve the plasticity that is required for development into hepatocytes. Direct differentiation may result from the exposure of competent cells to specific cues from the regenerating liver. The engrafted cells undergo epigenetic modifications, and consequently, the gene expression pattern is altered, leading to differentiation. Previous reports suggest that direct differentiation is the primary mechanism and can give rise to substantial regeneration of the liver (7–12% of the hepatocytes) by BM-MSCs. Sato *et al*. 52 examined the differentiation ability of human BM-MSCs into hepatocytes *in vivo* by directly inoculating them into livers with chronic damage because of alcohol exposure. In line with other findings 53, this strongly indicated that MSCs can differentiate into hepatocytes without undergoing fusion.

Cell fusion is the alternate pathway of plasticity in BM-MSCs. Fusion between BM-MSCs and hepatocytes has been demonstrated after irradiation of the host 53. The fused heterokaryons underwent a ploidy reduction to become normal hepatocytes, and hematopoietic myelomonocytic cells are the major source of hepatocyte fusion partners 54. However, cell fusion occurs at a very low frequency in normal adult physiological processes, and diseases resulting from extensive damage to the liver, such as chemical or viral induced hepatitis, may lack a sufficient number of viable cells for fusion events to take place. Recently, fusion between two hematopoietic cells has been observed in BM during the establishment of radiation chimeras, and this finding may explain why some investigators observed fusion between hepatocytes and donor cells and concluded that it was the principal mechanism by which hematopoietic cells acquired the hepatic phenotype 55.

### Immunomodulatory effects of MSCs to repress immune destruction

The immunomodulatory effects of MSCs have been extensively studied *in vitro* and *in vivo* 56–58. MSCs can suppress the activity of CD8^+^ cytotoxic T lymphocytes by inhibiting their proliferation directly and by increasing the relative proportion of CD4^+^ T lymphocytes indirectly 59. Because B-lymphocyte activation is largely T-cell dependent, the influence of MSCs on T lymphocytes may also indirectly suppress B-cell function 58. Meanwhile, MSCs are also capable of inhibiting the proliferation and antibody production of B cells *via* cell–cell contact and through the secretion of molecules such as interferon (IFN)-γ, interleukin (IL)-10, HGF, prostaglandin E2 (PGE2), TGF-β1, indoleamine 2,3-dioxygenase (IDO) and nitric oxide 56–58. In addition, MSCs efficiently inhibit the maturation, cytokine production and T-cell priming capacity of dendritic cells (DCs). The mechanism may involve the induction of mature DC differentiation, alteration of the actin distribution in DCs and the escape of DCs from an apoptotic fate 60,61. Furthermore, MSCs have a profound inhibitory effect on NK function, suppressing IL-2-induced cell proliferation, NK cytolytic activity, and the production of cytokines *via* the generation of soluble factors, including IDO and PGE2 62. Therefore, MSCs have attracted considerable interest in studies on immune-mediated therapies, and they have been proposed as cell therapies for degenerative, inflammatory and autoimmune diseases.

### Paracrine effect of MSCs

Similar to telocytes in liver regeneration 63,64, it has been reported that MSCs synthesize a wide variety of growth factors and cytokines that exert a paracrine effect on local cellular dynamics 65. Such paracrine effects include stimulation of revascularization and the enhancement of endogenous cell proliferation, leading to measurable therapeutic benefits in animal models of stroke, myocardial infarction, and renal failure independent of the direct differentiation of transplanted cells into the lineages of the respective tissues 66.

Using a model of chemically induced liver failure, the success and efficacy of MSC and MDH transplantation for the treatment of liver disease has been investigated 67. The results showed that both MSCs and MDHs differentiated into functional hepatocytes in the engrafted recipient liver and rescued liver failure. However, transplantation of MSCs had a greater rescue efficiency compared with MDHs. Furthermore, MSCs were found to be more resistant to oxidative stress *in vitro* and *in vivo*, which indicated that MSCs could protect cells against oxidative damage. Similar to other hepatotoxins, the hepatotoxicity of CCl_4_ is mediated by oxidative damage through metabolic activation, resulting in the production of free radicals and leading to lipid peroxidation, DNA damage and cellular death 68. MSCs enhanced the repopulation of necrotized tissue by stimulating the growth of endogenous cells rather than by protecting cells from necrosis. During *in vitro* co-culture, MSCs significantly promoted the proliferation and regeneration of murine hepatocytes after oxidative injury. This result suggests that differentiation of MSCs into hepatocytes was not the primary mechanism and that paracrine effects may contribute to the rescue of liver failure. The importance of the paracrine effects of MSCs was also demonstrated by Parekkaddan and his group. These investigators used MSC-derived molecules to successfully restore acute liver injury 69. Although they found evidence of paracrine effects upon MSCs transplantation and MSCs synthesize, a wide variety of growth factors and cytokines, the specific mechanism and the related molecular pathway still require further investigation.

### MSC inhibits hepatocellular apoptosis and stimulates liver regeneration

van Poll *et al*. 70 provided the first clear evidence that delivery of MSC-CM can dramatically reduce cell death and enhance liver regeneration in D-galactosamine-induced fulminant hepatic failure *in vivo* and *in vitro*. These authors reported that MSC-CM therapy led to a 90% reduction in apoptotic hepatocytes and a threefold increase in the number of proliferating hepatocytes *in vivo*. In addition, they detected a 4- to 27-fold increase in the expression of 10 genes known to be up-regulated during hepatocyte replication. In addition, the authors demonstrated that secretions from MSCs have a direct inhibitory effect on hepatocyte death and a stimulatory effect on proliferation in *ex vivo* assays. In addition, Du and colleagues found that rats that received reduced-size liver transplantation with MSC-CM infusion had significantly lower serum levels of tumour necrosis factor-α (TNF-α) and IL-1β compared with rats only receiving the medium treatment. Furthermore, on histological evaluation, they found that number of proliferating hepatocytes and sinusoidal endothelial cells in the MSC-CM treatment group had increased by 1.2- and 1.6-fold, respectively 71. It is believed that MSC secretions contain a number of trophic molecules, including soluble ECM glycoproteins, cytokines and growth factors 72. It remains unknown what specific mediators present in MSC-CM are responsible for the reduction in cell death and stimulation of liver regeneration. Previous studies demonstrated that several molecules are involved in this process 69, such as VEGF, TGF-β, TNF-α, HGF and IL-6.

## Problems and perspectives

Mesenchymal stem cells are easily accessible from a variety of tissues and that can contribute to liver regeneration, which makes MSCs an outstanding source for transplantation and offers a novel therapy for liver diseases. However, several problems must be considered.

First, the frequency of engraftment and differentiation of MSCs after transplantation remains unsatisfactory. To our current knowledge, only 1–3% of the host liver is readily repopulated by donor cells after cell transplantation 73. Similarly, the engraftment frequencies of transplanted donor cells in the recipient liver at 4 weeks post-transplantation was 4.4 ± 0.88% 74. Meanwhile, in an investigation of the hepatic ‘fate’ of transplanted MSCs in which MSCs were directly injected into the injured liver parenchyma of immunosuppressed rats, engraftment was limited to the site of injection, with an estimated differentiation efficiency lower than 0.5% at 4 weeks after transplantation 52. These studies indicate that *in vivo* differentiation of transplanted MSCs into HLCs represents a relatively rare and quantitatively unsatisfactory event.

Second, despite the important benefits arising from the use of MSC-based therapy, safety issues remain a concern, particularly regarding the long-term effects on immune function and the tumourigenic risk. Several studies suggest that MSCs might promote tumour growth *via* transformation, suppression of the antitumour immune response, and direct trophic action on tumour cells 75,76. Furthermore, MSCs could be recruited by cancer cells, and in turn they stimulated the migration and invasion of tumour cells through the secretion of angiogenic growth factors, including VEGF, FGF, platelet derived growth factor, and stromal-derived factor-1 77.

Third, MSCs in treating chronically injured liver may display a profibrogenic potential in chronic liver disease. Although many studies support the concept that MSCs play a positive role in the regeneration of cirrhotic liver 78, other studies have reported that MSCs significantly contribute to liver fibrosis by differentiating into pro-fibrogenic myofibroblast-like cells and provided convincing evidence that MSCs were predominant source of myofibroblasts 79. These issues and undesirable effects should be rationally addressed when applying MSCs for the treatment of liver diseases.

In addition to the above mentioned problems, the precise underlying mechanism of hepatic differentiation and the specific therapeutic effects of MSCs have not been fully elucidated. Further investigation is required to improve the efficacy and consistency of hepatic differentiation from MSCs and should address strategies to improve the long-term implantation of MSCs in the host liver. Regarding the mechanism of the therapeutic effect of MSCs, the paracrine effect of MSCs should be more thoroughly addressed. Further studies *in vitro* and *in vivo* are needed to achieve a better understanding of the paracrine effects of MSCs. Gene profiling of MDH-like cells revealed a complex interplay between cell receptors, signalling pathways and transcription factors that allow tissue cross-lineage conversion. A thorough molecular screening would provide key insights into the molecular mechanisms controlling the hepatic differentiation of MSCs. What is more, the clinical application of human stem cells in the treatment of liver disease is still in its infancy, and large clinical trials are needed to verify the therapeutic potential of MSCs in human liver disease.
